# The effects of *AQP4* rs162009 on resting‐state brain activity in Parkinson's disease

**DOI:** 10.1111/cns.14208

**Published:** 2023-04-10

**Authors:** Mengze Jiang, Yi Fang, Shaobing Dai, Xiaoli Si, Zhiyun Wang, Jiahui Tang, Ting Gao, Yi Liu, Zhe Song, Jiali Pu, Baorong Zhang

**Affiliations:** ^1^ Department of Neurology, Second Affiliated Hospital, School of Medicine Zhejiang University Zhejiang Hangzhou China; ^2^ Department of Anesthesiology, Women's Hospital, School of Medicine Zhejiang University Zhejiang Hangzhou China

**Keywords:** Aquaporin‐4, cognition, glymphatic system, Parkinson's disease, resting state functional MRI

## Abstract

**Background:**

We previously identified a significant association between Aquaporin‐4 (*AQP4*) and Parkinson's disease (PD).

**Objectives:**

To identify whether *AQP4* single‐nucleotide polymorphism (SNP) rs162009 affects regional brain activity and clinical phenotypes of PD.

**Methods:**

Low‐frequency fluctuation amplitude (ALFF) was used to evaluate spontaneous brain activity, regional homogeneity (ReHo) was used to evaluate the pace of activity of adjacent voxel regions, and degree centrality (DC) was used to describe the functional connection strength between a voxel and the whole brain. Disease severity and PD stage were assessed with the Movement Disorder Society‐sponsored revision of the Unified Parkinson's Disease Rating Scale and Hoehn and Yahr scales, and the Montreal Cognitive Assessment (MoCA) was used to assess the participants' cognitive function.

**Results:**

In patients with PD, *AQP4* SNP rs162009 was associated with a significant higher ALFF in the right caudate head and the left occipital gyrus, a significant lower ReHo in the right inferior frontal gyrus, a different DC in the right frontal gyrus, the left calcarine, and the right inferior temporal gyrus. A significant positive correlation between ALFF in the right caudate head and MoCA in rs162009_A carriers was found. A significant negative correlation between the DC at the left calcarine and MDS‐UPDRS and MDS‐UPDRS III in rs162009_A noncarriers was found.

**Conclusions:**

Our study further revealed the effect of *AQP4* SNP rs162009 on brain activity in PD, indicating that *AQP4* may play an important role in PD neuropathophysiology.

## Graphical abstract

AQP4 SNP rs162009 was associated with a significant higher ALFF in the right caudate head and the left occipital gyrus in patients with PD. There was a significant positive correlation between ALFF in the right caudate head and MoCA in rs162009_A carriers. AQP4 SNP rs162009 was associated with a significant lower ReHo in the right inferior frontal gyrus, a different DC in the right frontal gyrus, the left calcarine, and the right inferior temporal gyrus. A significant negative correlation between the DC at the left calcarine and MDS‐UPDRS and MDS‐UPDRS III in rs162009_A noncarriers was found.

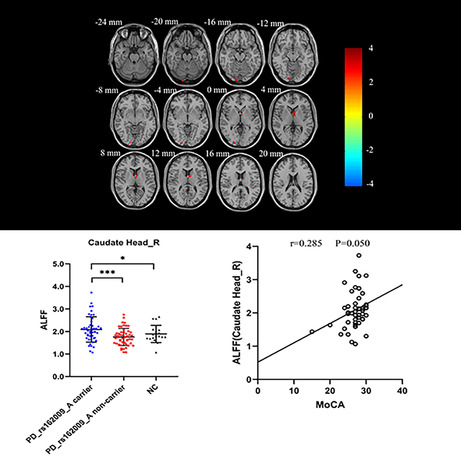

## 1 INTRODUCTION

Parkinson's disease (PD) is a common chronic neurodegenerative disease with a range of causes and clinical presentations.^1^ Patients with PD often show characteristic motor symptoms, such as tremor, stiffness, and slowness, and nonmotor symptoms, such as constipation, cognition, mood, and sleep disorder.^2^ The pathological features of PD include the aggregation of misfolded α‐Synuclein in the form of Lewy bodies, Lewy neurites, and neuronal and glial cytoplasmic inclusions,^3^ ultimately causing the death of major dopaminergic neurons in the substantia nigra and striatum.^4^ In addition to intracellular accumulation, α‐Synuclein can be secreted through unconventional exocytosis and spread in the extracellular environment.^5^, ^6^ Studies have shown that extracellular amyloid β (Aβ) and Tau aggregates, classic biomarkers of Alzheimer's disease, also play a role in PD^7^ and are related to a decline in cognitive ability.^8^ Therefore, the deposition of interstitial solutes is crucial.

Aquaporin‐4 (*AQP4*) is the main aquaporin expressed in the brain and is highly concentrated in perivascular astrocytic endfeet.^9^ Previous studies have shown that *AQP4* participates in the glymphatic system^10^ and plays an important role in the balance between cerebrospinal fluid and interstitial fluid.^11^ *AQP4* can also promote the clearance of water^12^ and waste proteins from the brain, including α‐Synuclein,^13^ Aβ,^14^ and Tau.^15^ An autopsy study of the aging human brain showed that the expression of *AQP4* in the neocortex of patients with PD was significantly negatively correlated with α‐Synuclein.^16^ The single‐nucleotide polymorphism (SNP) rs162009 is located between exons 0 and 1 of the *AQP4* gene and is assumed to be the promoter of the *AQP4*‐M23 splice variant. The frequency of *AQP4* rs162009_A was 0.561 in the populations and 0.560 in patients with PD. Previous research on *AQP4* rs162009 showed that rs162009_A was associated with PD, and carriers showed slower dementia conversion, better performance in alphanumeric number ordering and symbol digit modalities, and lower Aβ deposition in the putamen, anterior cingulum, and frontotemporal areas.^17^ Based on these findings, we speculated that *AQP4* rs162009 may play a role in the pathophysiological mechanism of PD. To our knowledge, there has been no study on the association between rs162009 and PD, except for our previous studies, and it is important to clarify the effect of this variation on the brain and its relationship with PD.

Neuroimaging genomics is a newly developed field, which integrates brain imaging and genetic data at the individual level to study how genetic risk factors affect brain phenotype changes.^18^ Genetic variation according to different location and context could affect cell function, which would be manifested as changes in brain structure and function. This strategy was applied to PD using fMRI and found that rs4680 of COMT gene, rs9468 of MAPT gene, rs429358 of APOE gene,^19^ and rs894278 of SNCA gene^20^ modulated different brain areas each associated with different cognitive domains. Therefore, individual genotyping and detection of brain activity change characteristics of PD might be used as a powerful tool for early diagnosis.

Amplitude of low‐frequency fluctuation (ALFF) is a resting state‐functional magnetic resonance imaging (rs‐fMRI) analysis method that can reflect the intensity of spontaneous neural activity in brain regions.^21^ Some studies have shown that changes in ALFF values in some brain regions can effectively distinguish PD patients from normal controls, which indicates that ALFF values can be used as a diagnostic biomarker of PD.^22^ Regional homogeneity (ReHo) was determined by the Kendall consistency coefficient (KCC) of the blood‐oxygen‐level‐dependent (BOLD) time‐series between a given voxel and its adjacent 26 voxels.^23^ The ReHo value represents the degree of local synchronization of brain activity, which can be regarded as an indicator of network centrality, and represents the importance of nodes in the functional connection groups in the brain.^24^ Previous studies have shown that ReHo values in some brain regions of patients with PD can be used as indicators to identify mild‐to‐moderate PD.^25^ Degree centrality (DC) is a voxel measurement used to evaluate the strength of functional connectivity of the whole brain, which represents global synchronization or global functional connectivity density.^26^ Studies have shown that DC is inhibited in brain regions related to cognition and movement in PD patients.^27^

In this context, we explored the relationship between the rs162009 polymorphism in the *AQP4* gene locus and PD and studied the changes in ALFF, ReHo, and DC in rs‐fMRI between specific genotypes (AA/AG and GG). In addition, we explored the relationship between *AQP4*‐regulated brain changes, cognitive function, and behavioral performance to determine the gene‐brain‐behavior relationship between PD patients with different gene phenotypes and normal controls.

## 2 MATERIALS AND METHODS

### 2.1 Participants

A total of 103 patients with PD and 20 normal controls (NCs) from the Parkinson's Progression Markers Initiative (PPMI) were enrolled. These participants were totally enrolled at nine PPMI sites. The participating PPMI sites were approved by an ethics standards committee before study initiation, and all individuals involved in the study obtained written informed consent. The PPMI study is an ongoing international multicenter cohort study aimed at identifying biomarkers of PD. Research protocols and methods are available on the PPMI website. Patients were recruited between 2010 and 2015 using strict inclusion and exclusion criteria. Inclusion criteria for PD patients were as follows^28^: (1) age ≥ 30 years old; (2) had no PD medications treatment; (3) the diagnosis was <2 years and Hoehn and Yahr (H&Y) stage I or II; (4) had at least two criteria of bradykinesia, rigidity, and resting tremor or either asymmetric resting tremor or asymmetric bradykinesia; (5) had a dopamine transporter (DAT) deficit on imaging; (6) had no dementia as determined by the site investigators. NCs were required to have: (1) no significant neurologic dysfunction; (2) no first‐degree family member with PD; and (3) a Montreal Cognitive Assessment (MoCA) score greater than 26. The criteria for exclusion were as follows: (1) no whole‐genome sequencing data and (2) no rs‐fMRI data. Demographic information and clinical characteristics are presented in Table 1.

**TABLE 1:** Clinical data of PD rs162009_A carriers, PD rs162009_A noncarriers and NCs.

Characteristics	PD rs162009_A carriers (*n* = 50)	PD rs162009_A noncarriers (*n* = 53)	NC (*n* = 20)	*p* ^‡^	*p* ^§^	*p* ^¶^
Age (years)	61.65 ± 9.19	61.41 ± 10.72	63.73 ± 9.85	0.448	0.103	0.089
Sex (male/female)	33/17	35/18	16/4	0.498	0.127	0.126
Age of onset	60.56 ± 9.15	60.03 ± 10.52	–	0.392	–	–
Course of disease	1.08 ± 0.99	1.38 ± 1.07	–	0.071	–	–
Education (years)	15.84 ± 2.72	15.42 ± 2.88	16.60 ± 2.54	0.222	0.143	0.055
MoCA	26.96 ± 2.73	26.91 ± 2.57	27.65 ± 1.53	0.459	0.146	0.114
MDS‐UPDRS total	35.20 ± 16.33	37.40 ± 18.30	–	0.261	–	–
MDS‐UPDRS I score	6.62 ± 4.72	7.36 ± 5.55	–	0.235	–	–
MDS‐UPDRS II score	7.00 ± 5.07	7.98 ± 5.18	–	0.167	–	–
MDS‐UPDRS III score	21.58 ± 11.29	22.06 ± 11.12	–	0.415	–	–
MDS‐UPDRS IV score	0.40 ± 1.32	0.60 ± 1.63	–	0.245	–	–
H‐Y scale	1.76 ± 0.48	1.70 ± 0.54	–	0.270	–	–

*Note*: After deleting the data with excessive head movement, there were 48 PD rs162009_A carriers, 52 PD rs162009_A noncarriers and 19 NCs left. Data are presented as mean ± standard deviation.

Abbreviations: H‐Y, Hoehn and Yahr; MDS‐UPDRS, Movement Disorder Society‐sponsored revision of the Unified Parkinson's Disease Rating Scale; MoCA, Montreal Cognitive Assessments; PD, Parkinson's disease.

^‡^ *p*, comparisons were made between PD rs162009_A carriers and PD rs162009_A noncarriers groups.

^§^ *p*, comparisons were made between PD rs162009_A carriers and NC groups.

^¶^ *p*, comparisons were made between PD rs162009_A noncarriers and NC groups.

### 2.2 Genotyping

Whole‐genome sequencing was performed using the PPMI database. Genomic DNA of the patients was extracted from whole blood according to the protocol described in the PPMI biologics manual. The Illumina TruSeq PCR Free DNA sample preparation kit was used to prepare the samples, and an Illumina HiSeq X Ten Sequencer was used to sequence the libraries. SNP pruning was performed using the PLINK software. Each participant was classified as either an A carrier or a noncarrier after genotyping. SNP rs162009_A did not deviate from the Hardy–Weinberg equilibrium (*p* > 0.05). As a result, 50 rs162009_A carriers and 53 noncarriers were included.

### 2.3 Clinical assessment

Disease severity and PD stage were evaluated in all participants using the Movement Disorder Society‐sponsored revision of the Unified Parkinson's Disease Rating Scale (MDS‐UPDRS) and the Hoehn and Yahr (HY) scale. Motor symptoms were assessed using the MDS‐UPDRS Part III and nonmotor symptoms were assessed using the MDS‐UPDRS Part I. Cognitive status was assessed using the Montreal Cognitive Assessment (MoCA).

### 2.4 MRI data acquisition

MRI data were collected using SIEMENS scanners, including Magnetom Trio Tim 3.0T, Magnetom Prisma 3.0T and Magnetom Verio 3.0T. The scanning sequences were as follows: (1) T1‐weighted axial image: sagittal scan, repetition time (TR) = 2300 ms, echo time (TE) = 3.0 ms, flip angle (FA) = 9.0°, matrix = 256 × 256, field of view (FOV) = 256 × 256 mm,^2^ slice thickness = 1.0 mm, slice number = 176, and voxel size = 1 mm^3^ isotropic. (2) rs‐fMRI scan: echo planar imaging pulse sequence, TR = 2400 ms, TE = 25.0 ms, FA = 80°, matrix size = 68 × 66, FOV = 240 × 240 mm^2^, slice thickness = 3.3 mm, slice number = 40, 210 volumes and voxel size = 3.3 mm^3^ isotropic.

### 2.5 MRI data preprocessing

MRI data were obtained using the DPARSF software (version 5.2, http://www.restfmri.net), which is based on SPM version 12 (https://www.fil.ion.ucl.ac.uk/spm/). The procedures used were as follows: the first 10 volumes of rs‐fMRI data from each participant were removed, and the remaining 200 volumes were corrected. Functional images were slice time‐corrected. Head motion was realigned after excluding data from participants whose head motion exceeded 3.0 mm or 3.0°. As a result, two rs162009_A carriers, one noncarrier and one NC were excluded. A total of 48 PD rs162009_A carriers, 52 PD rs162009_A noncarriers and 19 NCs were included in the follow‐up study. The structural and functional images were manually reoriented, including the rotation, translation, and original position of the head, to increase the accuracy of the registration, segmentation, and standardization. The T1 image was registered to the functional image, and we selected New Segment + DATEL to divide the image into gray matter, white matter, and cerebrospinal fluid and registered it to the Montreal Neurological Institute (MNI) space. For preprocessing of the ALFF calculation, we smoothed the image using a 4 × 4 × 4 full width at half maximum (FWHM) Gaussian kernel, removed the linear trend and nuisanced signals (white matter, cerebrospinal fluid signals, and head motion parameters calculated using rigid body six correction). For the preprocessing of the ReHo and DC calculations, we removed the linear trend and nuisanced signals (white matter, cerebrospinal fluid signals, and head motion parameters calculated using rigid body six correction), and performed band‐pass filtering (0.01–0.08 Hz).

### 2.6 ALFF analysis

To minimize low‐frequency drift and high‐frequency noise, we extracted the time series (0.01–0.08 Hz) and converted them to frequencies using a fast Fourier transform to obtain the power spectrum. The ALFF value is the square root of the power spectrum. We calculated the mean ALFF by dividing each voxel of the ALFF value by that of the whole brain. The ALFF values were compared between the different groups to obtain significant clusters. Finally, we calculated the ALFF values of clusters with significant differences and correlated them with clinical assessment scores such as the MDS‐UPDRS, MDS‐UPDRS I, MDS‐UPDRS III, MoCA, and HY.

### 2.7 ReHo analysis

We obtained a whole‐brain ReHo map by calculating the KCC between each voxel and its nearest voxels. We then calculated the mean KCC by dividing each voxel of the KCC value by the whole brain and smoothed it using a 4 × 4 × 4 FWHM Gaussian kernel. ReHo values were compared between different groups to obtain significant clusters. Finally, we calculated the ReHo values of clusters with significant differences and correlated them with clinical assessment scores such as the MDS‐UPDRS, MDS‐UPDRS I, MDS‐UPDRS III, MoCA, and HY.

### 2.8 DC analysis

We calculated the Pearson correlations between all voxel time series in the whole brain to obtain the whole‐brain functional connectivity matrix. We set the Pearson correlation coefficient >0.25 as the threshold to eliminate voxel counts with low temporal correlation. For each voxel, a map of degree was computed by counting the number of voxels to which they were connected. The Fisher Z transform was used for standardization, and a 4 × 4 × 4 FWHM Gaussian kernel was used to smoothen the DC maps. The DC values were compared between the different groups to obtain significant clusters. Finally, we calculated the DC values of clusters with significant differences and correlated them with clinical assessment scores such as the MDS‐UPDRS, MDS‐UPDRS I, MDS‐UPDRS III, MoCA, and HY.

### 2.9 Statistical analysis

Demographic and clinical data of rs162009_A gene carriers and noncarriers in patients with PD were analyzed using SPSS (version 25.0; https://www.ibm.com/cn‐zh/analytics/spss‐statistics‐software). We used a two‐sample t‐test or a chi‐square test. Continuous variables are expressed as mean ± standard deviation. The threshold for statistical significance was set at *p* < 0.05.

The MRI data between the groups were compared using SPM12, and the effect of genotype on rs‐fMRI images was analyzed using a two‐sample *t*‐test. Age, sex, and site were included as covariates. For brain regions with significant differences among different genotypes, we extracted the ALFF, ReHo, or DC values and analyzed the correlation between them and clinical data. The statistical significance threshold was defined as voxel‐level *p* < 0.01 with AlphaSim corrected cluster *p* < 0.05.

## 3 RESULTS

### 3.1 Demographic and clinical data

There were no significant differences in age, sex, age of onset, course of disease, education, MoCA, MDS‐UPDRS total, MDS‐UPDRS I, MDS‐UPDRS II, MDS‐UPDRS III, MDS‐UPDRS IV, or HY scale stage between groups (Table 1).

### 3.2 Effects of rs162009_A genotype on ALFF, ReHo, and DC between groups

**FIGURE 1:**
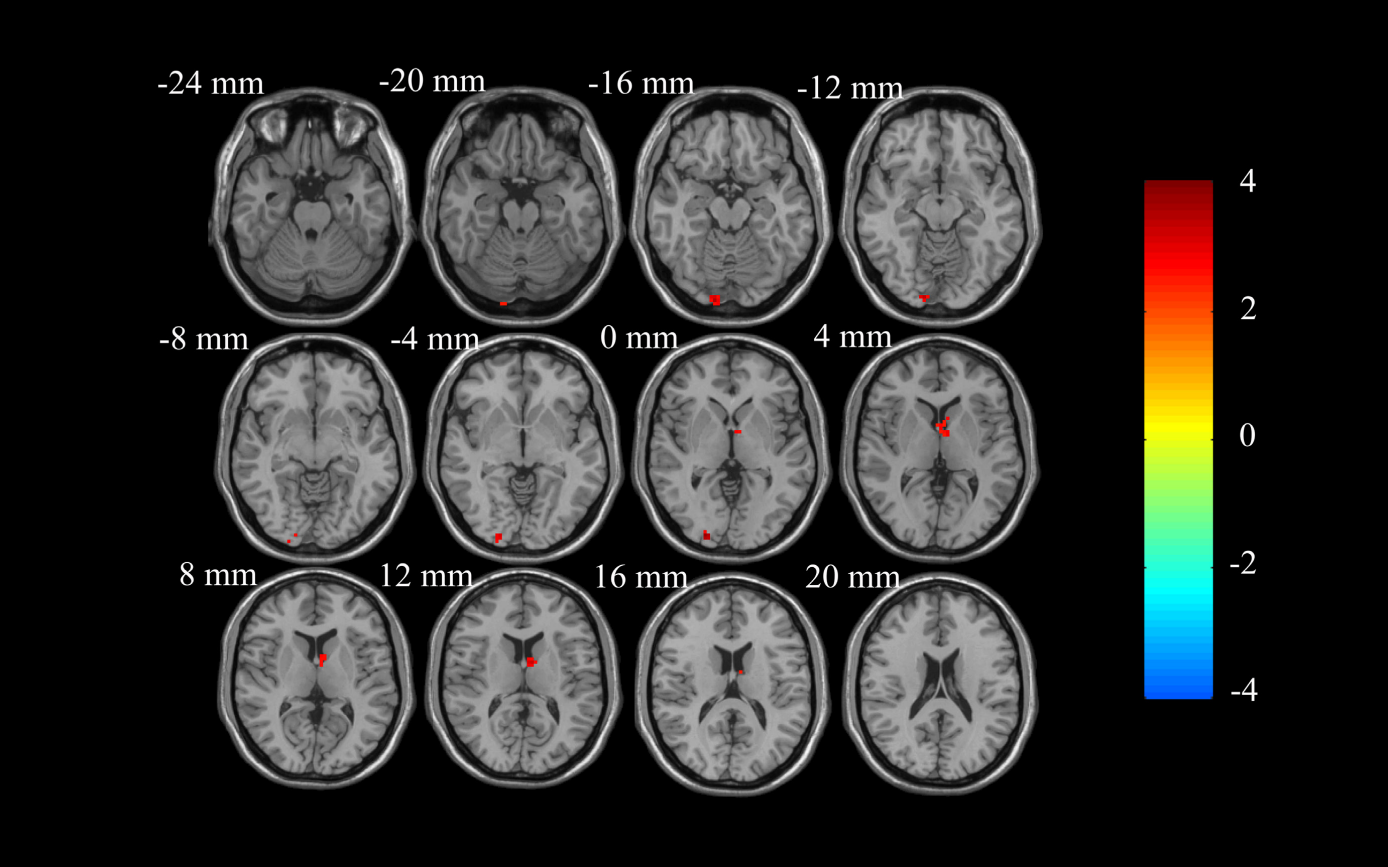
ALFF analysis of resting‐state brain activity for A carriers vs. non‐carriers of the rs162009 genotype in the PD group. ALFF at the right caudate head and the left middle occipital gyrus in rs162009_A carriers is higher than non‐carriers (voxel‐level *p* < 0.01, AlphaSim corrected cluster *p* < 0.05, with AlphaSim corrected cluster size >33 voxels). The color bar indicates the display window for the threshold *T*‐value maps. ALFF, amplitude of low‐frequency fluctuations; PD, Parkinson's disease.

A two‐sample t‐test analysis showed that in patients with PD, the rs162009_A genotype revealed higher ALFF at the right caudate head and the left middle occipital gyrus (Table 2, Figure 1). The ALFF values at the right caudate head and the left middle occipital gyrus in NCs were lower than those in PD rs162009_A carriers, but higher than those in PD rs162009_A non‐carriers (Figure 4A,B). Compared with NCs, PD rs162009_A carriers exhibited higher ALFF at the left inferior temporal gyrus and the right inferior frontal gyrus, opercular part, and lower ALFF at the calcarine fissure and surrounding cortex, the left superior parietal gyrus and left frontal lobe (Table 2, Figure S1A). Besides, PD rs162009_A noncarriers showed lower ALFF at the left lingual gyrus, the left middle occipital gyrus, and the left precentral gyrus (Table 2, Figure S1B).

**TABLE 2:** Significant clusters with the effects of rs162009_A genotype on ALFF, ReHo, and DC among patients with PD.

Effects	Brain regions	L/R	Number of voxels	Peak MNI coordinates			*T*‐value	*p*
				*x*	*y*	*z*		
ALFF	PD‐rs162009 vs.PD‐non‐rs162009							
	Caudate head	R	39	3	6	6	3.0624	<0.01
	Occipital_Mid (AAL)	L	37	−24	−93	0	3.6589	<0.01
	PD‐rs162009 vs. NC							
	Temporal_Inf (AAL)	L	46	−42	−39	−15	3.4139	<0.01
	Frontal_Inf_Tri (AAL)	L	52	−39	42	9	−4.5521	<0.01
	Frontal_Inf_Oper (AAL)	R	46	54	15	9	3.7002	<0.01
	Calcarine (AAL)	R	59	12	−90	9	−4.1949	<0.01
	Calcarine (AAL)	L	44	−3	−87	6	−3.6004	<0.01
	Parietal_Sup (AAL)	L	38	−24	−63	42	−4.9069	<0.01
	Frontal_Mid (AAL)	L	49	−39	15	45	−4.9519	<0.01
	PD‐non rs162009 vs. NC							
	Lingual (AAL)	L	160	−15	−96	−15	−5.254	<0.01
	Occipital_Mid (AAL)	L	204	−9	−99	3	−4.4921	<0.01
	Precentral (AAL)	L	50	−30	−3	57	−3.9575	<0.01
	Precentral (AAL)	L	43	−42	−12	54	−4.2073	<0.01
ReHo	PD‐rs162009 vs.PD‐non‐rs162009							
	Frontal_Inf_Oper (AAL)	R	104	48	12	36	−3.7627	<0.01
	PD‐rs162009 vs. NC							
	NONE							
	PD‐non‐rs162009 vs. NC							
	Fusiform (AAL)	R	93	36	−42	−24	−3.4017	<0.01
	Occipital_Sup (AAL)	R	422	21	−87	18	−4.8758	<0.01
DC	PD‐rs162009 vs.PD‐non‐rs162009							
	Frontal_Sup (AAL)	R	28	18	66	15	−4.02	<0.01
	Calcarine (AAL)	L	25	−6	−93	−9	4.1228	<0.01
	Temporal_Inf (AAL)	R	16	57	−54	−9	3.5585	<0.01
	Frontal_Inf_Tri (AAL)	R	14	−42	21	0	−3.55	<0.01
	PD‐rs162009 vs. NC							
	Temporal_Inf (AAL)	R	26	39	−3	−36	−4.246	<0.01
	Cerebelum_6 (AAL)	R	44	33	−42	−24	−4.0391	<0.01
	Supp_Motor_Area (AAL)	L	15	−6	6	75	−3.2595	<0.01
	Fusiform (AAL)	L	18	−33	−60	−15	−3.9301	<0.01
	Temporal_Sup (AAL)	R	14	57	−45	21	4.1676	<0.01
	Parietal_Sup (AAL)	L	26	−24	−69	42	−3.8196	<0.01
	Frontal_Mid (AAL)	L	17	−42	18	45	−3.789	<0.01
	PD‐non rs162009 vs. NC							
	Cerebelum_4_5 (AAL)	R	49	27	−45	−21	−3.5862	<0.01
	Frontal_Inf_Orb (AAL)	L	15	−24	15	−24	−4.1564	<0.01
	Occipital_Sup (AAL)	L	128	−9	−99	3	−4.5512	<0.01
	Lingual (AAL)	R	40	12	−51	3	−3.797	<0.01
	Fusiform (AAL)	R	30	24	−75	−9	−3.7148	<0.01
	Fusiform (AAL)	L	26	−30	−75	−12	−4.674	<0.01
	Fusiform (AAL)	L	33	−33	−63	−18	−3.891	<0.01
	Temporal_Pole_Sup (AAL)	L	14	−57	6	−6	−4.1102	<0.01
	Calcarine (AAL)	R	23	21	−99	0	−4.2411	<0.01
	Occipital_Mid (AAL)	L	19	−39	−72	0	−3.9104	<0.01
	Occipital_Mid (AAL)	R	18	24	−90	3	−3.7833	<0.01
	Occipital_Mid (AAL)	R	48	27	−87	15	−4.2526	<0.01
	Cingulum_Mid (AAL)	L	17	−9	−24	42	−4.0629	<0.01
	Frontal_Sup_Medial (AAL)	L	20	0	27	54	−3.8372	<0.01
	Postcentral (AAL)	L	15	−36	−21	48	−3.896	<0.01

*Note*: The *T*‐value denotes the statistical value of the peak voxel.

Abbreviations: AAL, Anatomical Automatic Labeling; ALFF, amplitude of low‐frequency fluctuations; DC, degree centrality; L, left; MNI, Montreal Neurological Institute; PD, Parkinson's disease; R, right; ReHo, regional homogeneity.

**FIGURE 2:**
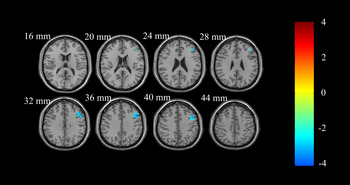
ReHo analysis of resting‐state brain activity for A carriers vs. non‐carriers of the rs162009 genotype in the PD group. ReHo at the right inferior frontal gyrus, opercular part in rs162009_A carriers is lower than non‐carriers (voxel‐level *p* < 0.01, AlphaSim corrected cluster *p* < 0.05, with AlphaSim corrected cluster size >91 voxels) . The color bar indicates the display window for the threshold *T*‐value maps. ReHo, regional homogeneity; PD, Parkinson's disease.

The PD rs162009_A genotype revealed lower ReHo at the right inferior frontal gyrus, opercular part (Table 2, Figure 2) than PD rs162009_A non‐carriers. The ReHo values at the right inferior frontal gyrus, opercular part in NCs were higher than those in PD rs162009_A carriers, but lower than those in PD rs162009_A noncarriers (Figure 4C). We also found that PD rs162009_A noncarriers revealed lower ReHo at the right fusiform gyrus and the right superior occipital gyrus than NCs (Table 2, Figure S2).

**FIGURE 3:**
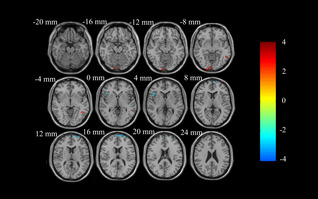
DC analysis of resting‐state brain activity for A carriers vs. noncarriers of the rs162009 genotype in the PD group. DC at the right superior frontal gyrus, dorsolateral, the left calcarine fissure and surrounding cortex, the right inferior temporal gyrus and the right inferior frontal gyrus, triangular part in rs162009_A carriers is lower than noncarriers (voxel‐level *p* < 0.01, AlphaSim corrected cluster *p* < 0.05, with AlphaSim corrected cluster size >12 voxels). The color bar indicates the display window for the threshold *T*‐value maps. DC, degree centrality; PD, Parkinson's disease.

**FIGURE 4:**
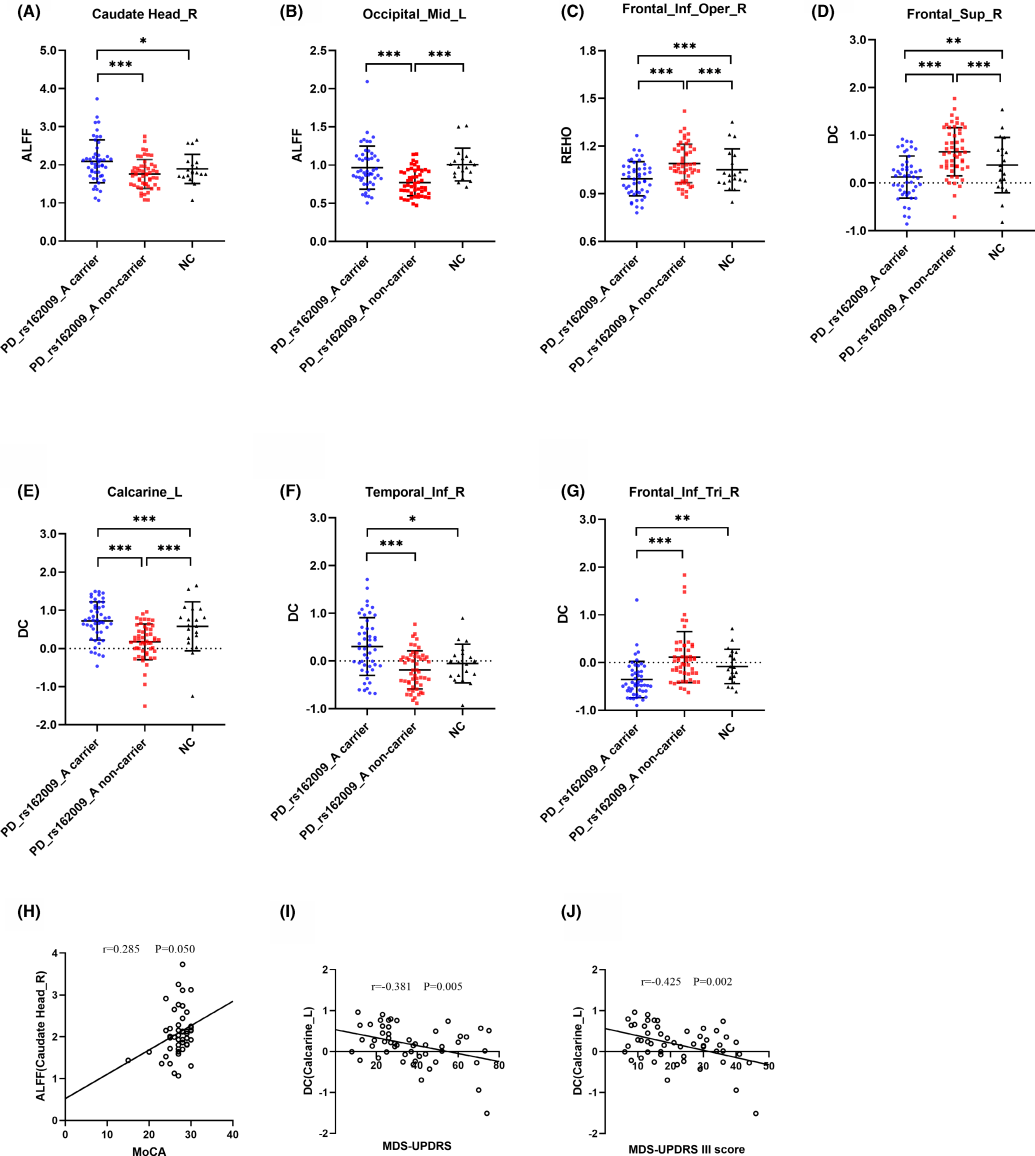
The effect of rs162009_A genotype in PD patients and NCs. (A) and (B) Scatter plot for the ALFF comparison of PD rs162009_A carriers, noncarriers and NCs at the right caudate head and the left middle occipital gyrus. (C) Scatter plot for the ReHo comparison of PD rs162009_A carriers, non‐carriers and NCs at the right inferior frontal gyrus, opercular part. (D–G) Scatter plot for the DC comparison of PD rs162009_A carriers, non‐carriers and NCs at the right superior frontal gyrus, dorsolateral, the left calcarine fissure and surrounding cortex, the right inferior temporal gyrus and the right inferior frontal gyrus, triangular part. (H) Correlation between ALFF and MoCA at the right caudate head in rs162009_A gene carriers. (I, J) Correlation between DC and MDS‐UPDRS, MDS‐UPDRS III score at the left calcarine fissure and surrounding cortex in rs162009_A gene noncarriers. Scores of MoCA, MDS‐UPDRS, and MDS‐UPDRS III for the X‐axis and ALFF, DC values for the Y‐axis. ALFF, amplitude of low‐frequency fluctuations; Calcarine, calcarine fissure and surrounding cortex; DC, degree centrality; Frontal_Inf_Oper, inferior frontal gyrus, opercular part; Frontal_Inf_Tri, inferior frontal gyrus, triangular part; Frontal_Sup, superior frontal gyrus, dorsolateral; MDS‐UPDRS, Movement Disorder Society‐sponsored revision of the Unified Parkinson's Disease Rating Scale; MoCA, Montreal Cognitive Assessment; Occipital_Mid, middle occipital gyrus; PD, Parkinson's disease; ReHo, regional homogeneity; Temporal_Inf, inferior temporal gyrus. ***p < 0.001. **p < 0.01. *p < 0.05.

In patients with PD, the rs162009_A genotype revealed lower DC values at the right superior frontal gyrus and the right inferior frontal gyrus, triangular part, and the higher DC values at the left calcarine fissure and surrounding cortex, the right inferior temporal gyrus (Table 2, Figure 3). The DC values at the right superior frontal gyrus and the right inferior frontal gyrus, triangular part, were higher in NCs than in PD rs162009_A carriers, but lower than in PD rs162009_A non‐carriers (Figure 4D,G). The DC values at the left calcarine fissure and surrounding cortex, as well as at the right inferior temporal gyrus, were lower in NCs than in PD rs162009_A carriers, but higher than in PD rs162009_A non‐carriers (Figure 4E,F). The differences between PD rs162009_A carriers and NCs were mainly reflected in temporal lobe, superior cerebellum, left fusiform gyrus, left superior parietal gyrus, and left middle frontal gyrus (Table 2, Figure S3A). Compared with NCs, PD rs162009_A noncarriers exhibited lower DC at the superior cerebellum, fusiform gyrus, occipital lobe, temporal lobe, right lingual gyrus, right calcarine fissure and surrounding cortex, left median cingulate and paracingulate gyri, frontal lobe, and postcentral gyrus (Table 2, Figure S3B).

### 3.3 Correlation analysis

The correlation between ALFF, ReHo, and DC in significantly different brain regions, cognition, and behavior was examined in rs162009_A gene carriers and noncarriers with PD. For PD rs162009_A allele carriers, the ALFF of the right caudate head was significantly positively correlated with the MoCA (*r* = 0.285, *p* = 0.050; Figure 4H). For PD rs162009_A noncarriers, the DC of the left calcarine fissure and surrounding cortex was significantly negatively correlated with the MDS‐UPDRS (*r* = −0.381, *p* = 0.005; Figure 4I) and MDS‐UPDRS III (*r* = −0.425, *p* = 0.002; Figure 4J).

## 4 DISCUSSION

To our knowledge, this is the first study to investigate the effect of *AQP4* rs162009 in patients with PD. Our results showed that in rs162009_A carriers with PD, the ALFF value of the right caudate head and the left middle occipital gyrus was higher than that in noncarriers, the ReHo value of the right opercular inferior frontal gyrus was lower than that in noncarriers, the DC value of the right dorsolateral superior frontal gyrus and the right triangular inferior frontal gyrus was lower than that in noncarriers, and the DC value of the left calcarine fissure and surrounding cortex, and the right inferior temporal gyrus was higher than that in noncarriers. We further revealed a significant positive correlation between the ALFF and MoCA in the right caudate head, a significant negative correlation between the DC and MDS‐UPDRS and MDS‐UPDRS III at the left calcarine fissure and surrounding cortex. These findings provide evidence to support the view that *AQP4* rs162009 affects spontaneous brain activity in patients with PD.

Caudate dopaminergic dysfunction is considered to be the pathophysiological basis of PD^29^ and is related to the common symptoms of PD, such as cognitive impairment,^30^ depression,^31^ working memory,^32^ rapid eye movement sleep behavior disorder,^33^ and gait problems.^34^ Animal studies show that *AQP4* knockout will lead to obstruction of interstitial fluid clearance and accumulation of interstitial fluid in the cortex and caudate nucleus, resulting in a decrease in tortuosity and an increase in the volume fraction and molecular diffusion rate in the extracellular space within the caudate nucleus.^35^ Studies on the volume of gray matter have shown that the volume of the bilateral caudate in patients with PD is reduced.^36^ Rs‐fMRI was used in this study. Previous studies found that the ALFF and ReHo values in the caudate in the PD group changed.^37^, ^38^ Studies have shown that the functional connectivity of the caudate nucleus head network in patients with PD receiving drug treatment was significantly improved.^39^ We found that the ALFF value of rs162009_A carriers with PD at the right caudate head was higher than that of noncarriers, which provided evidence that *AQP4* might have some protective effects on the function of the right caudate head in PD patients, such as brain interstitial fluid transportation, and could change brain activity in PD.

Previous studies have shown that patients with PD and mild cognitive impairment have progressive atrophy of the caudate,^40^ and Mini‐Mental State Examination scores are significantly correlated with the level of caudate head atrophy.^41^ Early abnormal binding of dopamine transporters in the caudate nucleus is associated with an increased risk of cognitive impairment, depression, and gait problems in the next four years.^42^ Right caudate nucleus volume is a significant independent predictor of conversion to mild cognitive impairment in patients with PD.^41^ We found that in patients with PD, the ALFF value of rs162009_A carriers at the right caudate head was higher and positively correlated with MoCA, indicating that there was a correlation between the ALFF value in this region and cognition. Previous studies have shown that *AQP4* rs162009_A carriers have slower dementia conversion,^17^ and our results may explain this phenomenon to a certain extent.

Besides, we found that PD rs162009_A carriers exhibited higher ALFF at the left inferior temporal gyrus and the right inferior frontal gyrus, opercular part, and lower ALFF at the calcarine fissure and surrounding cortex, the left superior parietal gyrus, and left frontal lobe. PD rs162009_A noncarriers showed lower ALFF at the left lingual gyrus, the left middle occipital gyrus, and the left precentral gyrus. It was similar to the results of previous functional magnetic resonance studies of PD.^43^

The frontal cortex is typically considered to be associated with cognitive and executive functions.^44^ It has been reported that patients with PD exhibit changes in frontal cortex thickness^45^ and lower gray matter volume.^46^ Compared with patients without cognitive impairment, the volume of frontal gray matter^47^ and metabolism of the frontal lobe^48^ decreased in patients with dementia. The ReHo value of the left superior frontal gyrus in early onset PD patients is higher than that in late‐onset PD patients.^49^ An rs‐fMRI study revealed DC value changes in the right frontal lobe of PD patients.^50^ Our results showed that rs162009_A was associated with a lower ReHo value in the right opercular inferior frontal gyrus and DC value in the right dorsolateral superior frontal gyrus and the right triangular inferior frontal gyrus. This may be related to the lower amyloid load in the frontal cortex of *AQP4* rs162009_A carriers.^17^ Autopsy study showed that the loss of *AQP4* location around the frontal lobe vessels was related to increased Aβ load.^51^ However, our study did not find a correlation between α‐Synuclein, Aβ, Tau, ReHo, and DC in the frontal lobe of rs162009_A carriers. The causes of the low amyloid deposition in the frontal lobe of these patients require further study.

Calcarine fissure and surrounding cortex is a part of the occipital lobe, where the primary visual cortex is concentrated. PD patients had lower gray matter and white matter volumes in calcarine,^52^ and lower nodal efficiency of the calcarine area.^53^ Previous studies found that compared with the control group, PD patients presented lower DC value in calcarine area.^54^ We found that the DC value of the left calcarine fissure and surrounding cortex was higher than that in noncarriers, which explained the protective effect of *AQP4* rs162009 in part. It was worth noting that DC at the left calcarine fissure and surrounding cortex of PD rs162009 gene noncarriers were negatively correlated with the score of motor symptoms, which was similar to the previous PD study.^55^ However, there are few reports on the role of *AQP4* in these regions, so its mechanism needs to be further explored.

Although the results were reliable, our study had some limitations. First, although there was no significant difference in the course of disease between rs162009_A carriers and noncarriers in patients with PD, the course of disease in noncarriers was greater than that in carriers, which may have interfered with the results. Second, there were few normal controls with rs‐fMRI images and genotyping data in the PPMI database, which cannot be matched with patients with PD. Therefore, this study did not examine the interaction between disease and genotype. Our study did not genotype the NC group, but compared it with PD rs162009_A carriers and noncarriers as a single group to clarify whether rs162009_A carriers or noncarriers show more abnormal in their brain activity. Finally, the current findings should be replicated using larger sample numbers.

## 5 CONCLUSION

In conclusion, this study provides preliminary evidence for *AQP4* genotype‐related changes in brain function in patients with PD and helps to better understand the neurobiological mechanisms of PD.

## AUTHOR CONTRIBUTIONS

Mengze Jiang, Xiaoli Si, Ting Gao, and Jiali Pu developed and designed the study concept. Mengze Jiang collected the data. Yi Fang and Zhiyun Wang conducted correlation analysis. Yi Liu, Zhe Song, Shaobing Dai, and Baorong Zhang provided guidance for this study. Mengze Jiang wrote the first draft of this study and completed the revision with Jiali Pu′s help. All authors have approved the final article.

## FUNDING INFORMATION

This study was supported by the National Natural Science Foundation of China (Nos. 82001346 and 82001353) and the Key Research and Development Program of Zhejiang Province (No. 2020C03020).

## CONFLICT OF INTEREST STATEMENT

The authors declare no competing interests.

## Supporting information


Figure S1.

Figure S2.

Figure S3.

## Data Availability

Data used in the preparation of this article were obtained from the Parkinson's Progression Markers Initiative (PPMI) database (www.ppmi‐info.org/data). For up‐to‐date information on the study, visit www.ppmi‐info.org.
