# Supplementary figures and images for: The effects of AQP4 rs162009 on resting‐state brain activity in Parkinson's disease
Source: CNS Neurosci Ther. 2023 Apr 10;29(9):2645–55. doi: 10.1111/cns.14208 (PMC10401089; doi:10.1111/cns.14208)

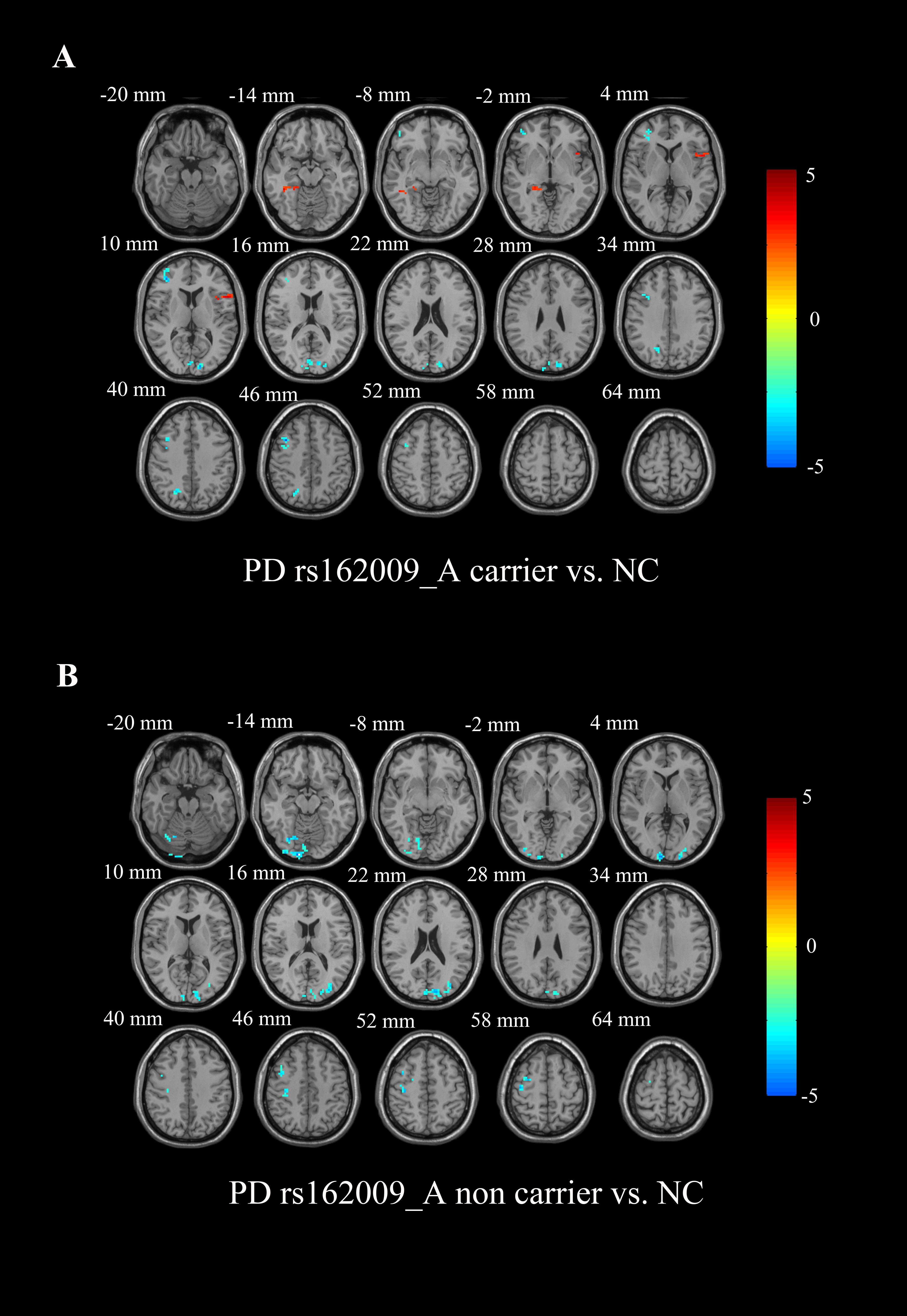

Supplement: Supplementary file 1 — Figure S1. [file CNS-29-2645-s001.tif]

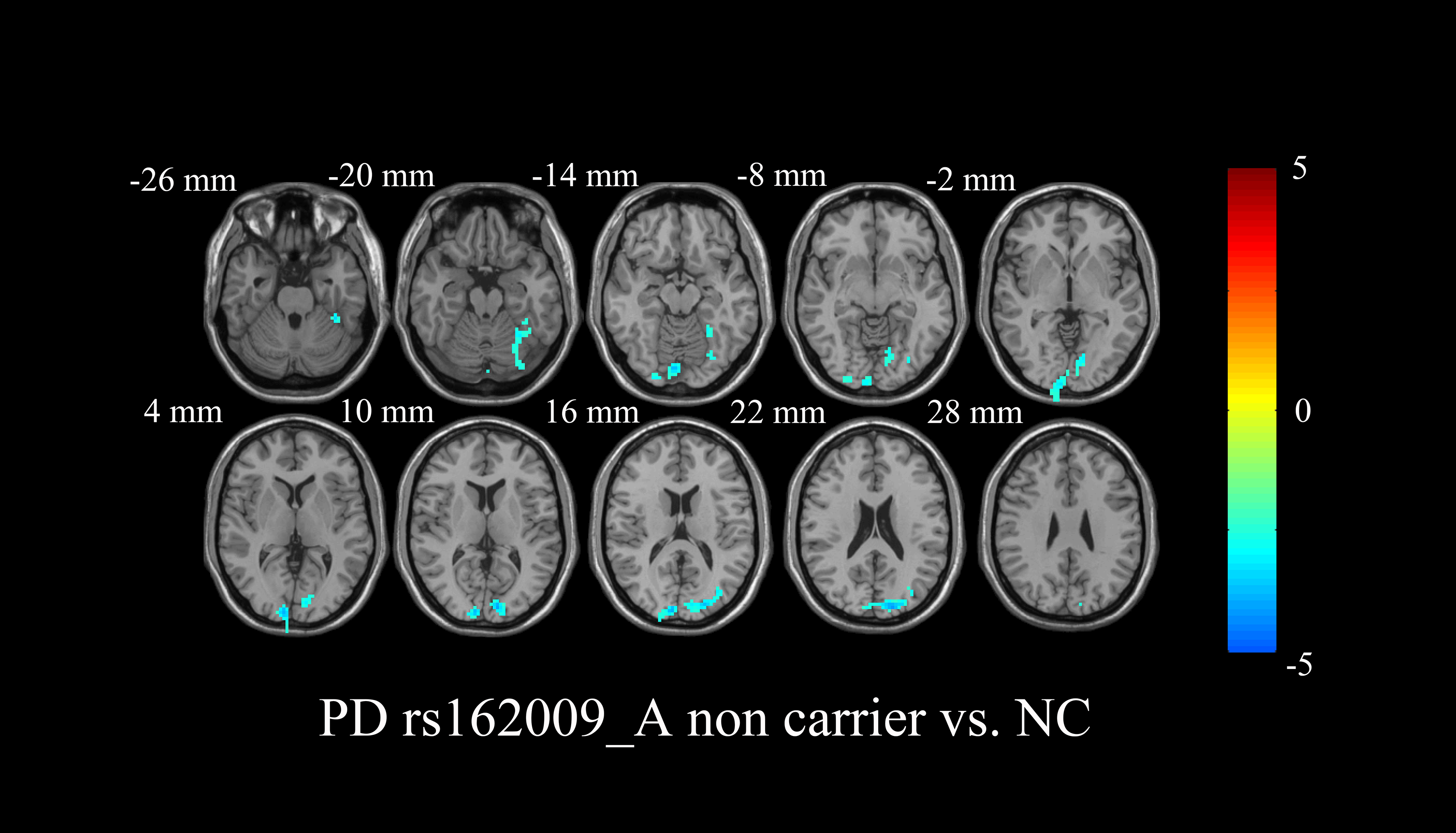

Supplement: Supplementary file 2 — Figure S2. [file CNS-29-2645-s003.tif]

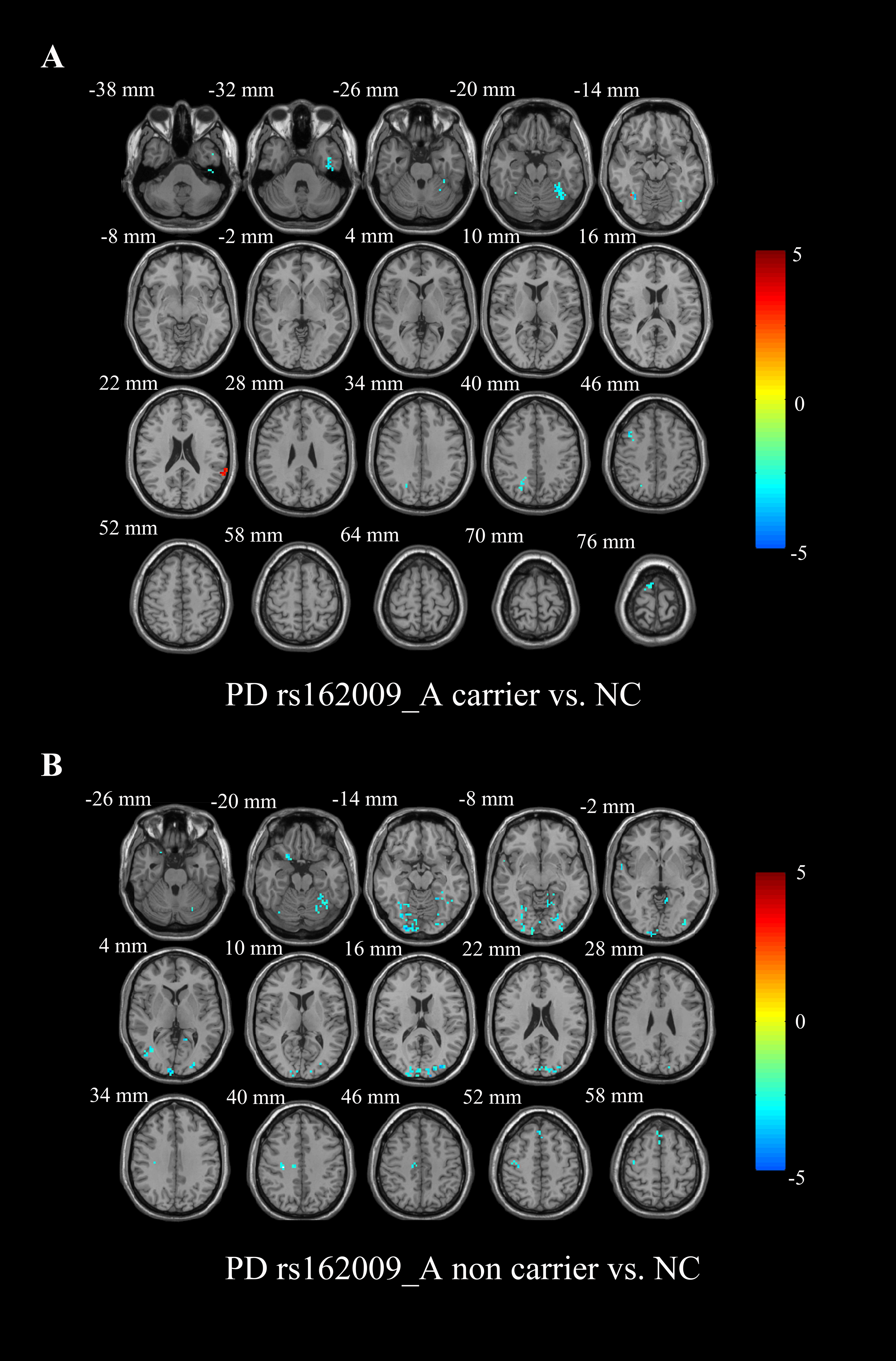

Supplement: Supplementary file 3 — Figure S3. [file CNS-29-2645-s002.tif]
